# Navigating Epstein–Barr virus autoimmunity: role of NK cells and T cells in multiple sclerosis

**DOI:** 10.1038/s41392-024-01774-8

**Published:** 2024-02-29

**Authors:** Chu Xie, Cong Sun, Mu-Sheng Zeng

**Affiliations:** State Key Laboratory of Oncology in South China, Guangdong Provincial Clinical Research Center for Cancer, Guangdong Key Laboratory of Nasopharyngeal Carcinoma Diagnosis and Therapy, Sun Yat-sen University Cancer Center, Guangzhou, P. R. China

**Keywords:** Infection, Rheumatic diseases

In a recent study published in *Cell*, Vietzen and colleagues discovered the novel role of natural killer (NK) cells and EBV-specific CD8^+^ T cell response in controlling EBV-inducing autoimmune response and collectively defined virus and host genetics factors associated with a significantly raised risk of multiple sclerosis.^1^

Multiple sclerosis (MS) is a central nervous system (CNS) autoimmune disease affecting approximately 2.8 million people globally. Various host factors and pathogens have been suspected as triggers of MS, and significant progress has been achieved recently in understanding the pathogenesis of multiple sclerosis.^2^ A large-scale epidemiological study published last year found a 32-fold increase in the risk of MS following Epstein–Barr virus (EBV) infection, compellingly supporting a leading role of EBV infection in MS development.^3,4^ Another study in the same year showed that EBV EBNA1 protein-derived peptide, EBNA_386–405_ could elicit cross-reactive antibodies against host CNS protein GlialCAM_370–389_ and induce MS-like pathology in mouse model.^5^ However, as EBV universally infects over 90% of the population with only a small portion developing MS, and certain healthy individuals also exhibit EBNA_386–405_-specific immune responses, then arises the question of what additional factors could protect people from MS? The latest study reveals that specific subgroups of NK cells and T cells play a critical role in controlling autoimmunity to prevent MS.

**Fig. 1:**
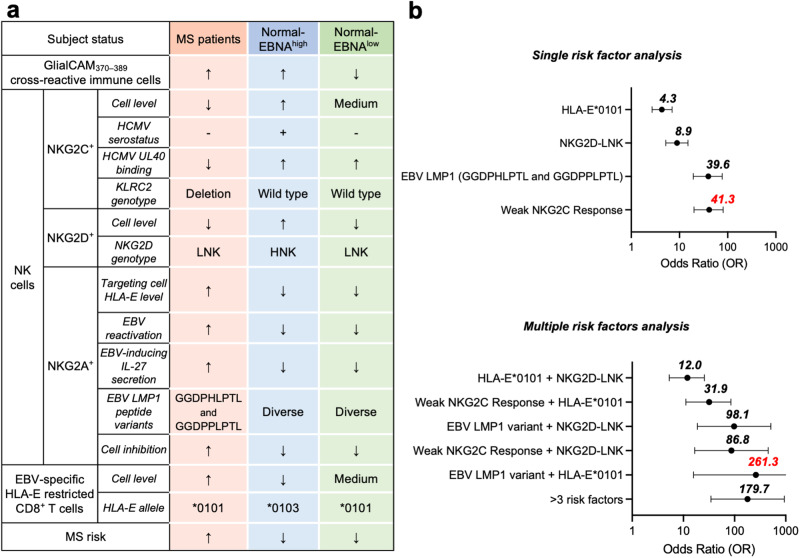
Summary table and risk factor analysis of the study. **a** Two hundred and seventy EBNA-1 seropositive MS patients and 270 matched EBV-seropositive healthy control according to sex, age, time since EBV seroconversion and occurrence of IM were recruited. While EBNA^high^ healthy controls were found with equally high levels of EBNA1-GlialCAM cross-reactive antibody, NK cells and EBV-specific CD8^+^ T cells were identified as critical in MS occurrence, and multiple virus and host genetics factors were discovered influencing NK and T cell function. **b** After statistical analysis, weak NKG2C response as an individual factor and EBV LMP1 variant with HLA-E*0101 as a multiple factor group were found highly associated with increased MS risk by 41-fold and 261-fold. The summary table is colored to identify different subject groups. The original data of risk factor analysis came from the reported research article and mean odds ratios for each factor or factor combination group were marked beside with red text indicating the largest value in each chart. The icon ↑↓ indicated the high or low factor level and the icon + and − indicated the positive or negative factor level

In this study, Vietzen et al. recruited EBNA1-seropositive MS patients and a matched control cohort of healthy individuals and further stratified the healthy controls into EBNA^high^ or EBNA^low^ group according to EBNA_386–405_ antibody level (Fig. 1a). This enables them to investigate variables that promote or mitigate MS development in people with the potentially pathogenic EBNA_386–405_-specific antibodies. Compared to EBNA^low^ controls, both MS patients and EBNA^high^ controls were found to have similar elevated levels of cross-reactive antibody, and the authors speculated that efficient suppressive factors of MS existed in the EBNA^high^ group. With the comparison of MS patients and the EBNA^high^ controls, they identified that NKG2C+ and NKG2D+ NK cells were effector cells controlling autoimmune responses against GlialCAM_370–389_. Host genetic factors (KLRC2 genotype for NKG2C+ NK cells and NKG2D genotype for NKG2D+ NK cells affecting NK cell activation and proliferation) and virus-related factors (Human Cytomegalovirus [HCMV] infection and HCMV UL40 variants affecting NKG2C+ NK cell response) could mediate NK cell function and thus influencing the risk of MS. Meanwhile, EBV-inducing IL27 secretion and certain expression of EBV LMP1 variants (GGDPHLPTL and GGDPPLPTL) could upregulate HLA-E on GlialCAM_370–389_-specific immune cell to evade the NKG2A+ NK cell cytotoxic effects. Generally, factors associated with impaired NK cell function or enhanced evasion of HLA-E-presenting autoreactive immune cells from NK cytotoxicity were positively correlated to MS. This underscores the central role of NK cells in controlling the EBV-induced autoimmunity and preventing MS pathogenesis.

Besides, Vietzen et al. found that EBV-specific HLA-E-restricted CD8^+^ T cells could be a protective factor against MS. As EBV-infected GlialCAM_370–389_-specific B cells were found to drive MS development, the EBV-specific HLA-E-restricted CD8^+^ T cell subgroup actively kill this specific group of B cells, and the host HLA-E*0103/0103 genotype was found connecting with even stronger cytotoxic effect. Both the cell level of EBV-specific HLA-E-restricted CD8^+^ T cells and the ratio of HLA-E*0103/0103 genotype are significantly higher in the EBNA^high^ controls than MS patients or EBNA^low^ controls, suggesting a protective role of this EBV-specific CD8^+^ T cell subgroup.

As specific subgroups of NK cells and CD8^+^ T cells were identified suppressing the GlialCAM_370–389_-specific autoimmunity and various host genetic or infectious factors could impair their function and influence the risk of MS, to evaluate how these factors contributing to the risk of MS, Vietzen et al. comprehensively analyzed the individual or group contributions of factors to MS in this study (Fig. 1b). They found that among the individual factors, weak NKG2C response was associated highest risk of MS (odds ratio (OR) with 95% confidence interval (CI), 41.3 95% CI 20.0–80.4). With different factors displaying complex interplay, multiple factors combined were further analyzed and among them EBV LMP1 variant (GGDPHLPTL and GGDPPLPTL) plus HLA-E*0101 allele together were found associated with a 261.3-fold (OR with 95% CI, 261.3 95% CI 15.8–431.0) increase in the risk of developing MS for people with high EBNA_386–405_-specific antibody level.

Overall, this study provides us with valuable insights into how NK cells and CD8^+^ T cells are mediated by virus and host genetics factors in regulating EBV-inducing autoimmunity. These findings could enhance the early and accurate identification of individuals at high risk of MS, enabling prompt monitoring and intervention of MS at an early stage. It would be inspiring to clarify whether the activation of specific NK cells or boost of EBV-specific HLA-E-restricted CD8^+^ T cells would be an effective therapeutic approach for MS. Additionally, besides GlialCAM, EBV EBNA1 protein has also been reported to elicit an autoimmune response through molecular mimicry of other CNS proteins such as ANO2 and CRYAB. This study would shed light on a parallel study for the role of NK cells, along with EBV-specific CD8^+^ T cells in mediating different antigen-specific autoimmune responses in MS development. Nevertheless, this study gives significant insight into the answer to different responses to EBV-inducing autoimmunity among the population with equal level cross-reactive GlialCAM antibody, which facilitates accurate prediction of MS occurrence and development of novel therapy against MS in the future.
